# Risk prediction tool for use and predictors of duration of postoperative oxygen therapy in children undergoing non-cardiac surgery: a case-control study

**DOI:** 10.1186/s12871-018-0595-4

**Published:** 2018-11-02

**Authors:** Maliwan Oofuvong, Siriwimol Ratprasert, Thavat Chanchayanon

**Affiliations:** Department of Anesthesiology, Faculty of Medicine, Prince of Songkla University, 15 Kanjanavanich Road, Songkhla, 90112 Thailand

**Keywords:** Predictors, Duration of use, Postoperative oxygen therapy, Pediatric anesthesia

## Abstract

**Background:**

We aimed to construct a prediction tool for postoperative oxygen therapy and determine predictors of duration of use among children undergoing non-cardiac surgery.

**Methods:**

Data from this case-control study was obtained from a database of 9820 children aged < 15 years who underwent general anesthesia between January 2010 and December 2013 at a tertiary care hospital in southern Thailand. The primary outcomes were the use and duration (hours) of postoperative oxygen therapy (cases). Cases were matched with controls on age group and year of surgery in a ratio of 1:4. A negative binomial hurdle model was used to obtain significant predictors of any use and number of hours of oxygen therapy. A risk score was derived from the coefficients of the significant predictors. The risk score, adjusted odds ratio (OR) for any use and count ratio (CR) for duration of postoperative oxygen therapy and 95% confidence interval (CI) were determined.

**Results:**

A total of 288 cases and 1152 controls were included. The median (inter-quartile range) duration of oxygen therapy delivered was 17 (9–22) hours. An optimal risk score for predictors of oxygen use was 12 (0–32) giving an area under the receiver operating characteristic curve of 0.93. Predictors of high risk need for oxygen therapy (score ≥ 12) were thoracic surgery (OR = 278, 95% CI = 44.6–1733) and having desaturation perioperatively (OR = 459.8, 95% CI = 169.7–1246). Intermediate risk factors (score 8–11) were having bronchospasm (OR = 92.4, 95% CI = 29.7–287.5) and upper airway obstruction/laryngospasm (OR = 61.5, 95% CI = 14.4–262.4) perioperatively. Significant predictors of duration of oxygen therapy were probably difficult airway (CR = 2.2, 95% CI = 1.4–3.5), history of delayed development (CR = 2.3, 95% CI = 1.5–3.6), airway (CR = 3.0, 95% CI = 1.6–5.8), orthopedic (CR = 2.1, 95% CI = 1.1–4.3), thoracic (CR = 4.9, 95% CI = 2.3–10.1) and abdominal surgery (CR = 4.2, 95% CI = 2.1–8.1), compared to eye surgery.

**Conclusions:**

Our risk prediction tool for the use of postoperative oxygen therapy provided a high predictive ability. Children who have thoracic surgery, desaturation, bronchospasm, upper airway obstruction or laryngospasm will most likely need postoperative oxygen therapy, regardless of other factors, while those with a probably difficult airway, history of delayed development, or thoracic/abdominal surgery will most likely need longer duration of oxygen therapy.

**Electronic supplementary material:**

The online version of this article (10.1186/s12871-018-0595-4) contains supplementary material, which is available to authorized users.

## Background

At the post-anesthetic care unit (PACU), postoperative oxygen therapy is often given to patients, particularly children, after finishing general anesthesia. Studies have shown that general anesthesia, which induces airway closure, ventilation, and perfusion mismatch, can decrease the functional residual capacity causing hypoxemia and necessitates supplemental oxygen during the first postoperative hour that continues into the postoperative period [1–5]. Since the prevalence of hypoxemia at the PACU in children can be as high as 21% [5], supplemental oxygen therapy during transfer to the PACU [6] and throughout the PACU stay is recommended [7].

Despite the usefulness of oxygen therapy, longer use of postoperative oxygen therapy can be harmful to children [8]. In addition, surgery-related or anesthesia-related factors may also be important. However, a risk prediction tool for the need of postoperative oxygen therapy in children has never been developed and predictors of the duration and use of postoperative oxygen therapy in children have not been determined. Therefore, the objectives of this study were to develop a risk prediction tool for postoperative oxygen therapy and to determine predictors of duration of use in children undergoing non-cardiac surgery with general anesthesia. The results of this study may help guide anesthesia personnel determine which patient groups are more likely to require postoperative oxygen therapy as well as those who may require longer use.

## Methods

This was a matched case-control study among children who underwent non-cardiac surgery at Songklanagarind Hospital which is an 853-bed tertiary care university hospital in southern Thailand. The study was approved by the Institutional Ethics Committee of the Faculty of Medicine, Prince of Songkla University, Songkhla, Thailand (Chairperson Assoc. Prof. Boonsin Tangtrakulwanich) on October 8, 2014 [EC57294081].

### Participants

The surveillance anesthetic database under quality assurance conference by nurse anesthetists performed every 2 months was used to identify children aged less than 15 years who received postoperative oxygen therapy after undergoing non-cardiac surgical procedures as well as digital imaging or other interventions under general anesthesia with or without regional anesthesia or peripheral nerve block between January 2010 and December 2013. All identified children were confirmed eligible by chart review of electronic anesthetic records by two investigators (MO, SR). Patients were excluded if they were classified as American Society of Anesthesiologists (ASA) physical status 4 or 5, had preoperative oxygen saturation by pulse oximetry (SpO_2_) < 95% at room air, received preoperative oxygen therapy, were endotracheally intubated, or their lungs were mechanically ventilated or both before or after surgery, had congenital cyanotic heart disease, or had cardiac surgery. These criteria were used to exclude pre-existing active respiratory problems before surgery or cardiopulmonary problems after surgery. The children who needed postoperative mechanical ventilation have more severe respiratory insufficiency and may have different risk factors compared to children who need only postoperative oxygen and were therefore excluded from the study.

### Anesthetic practice and standard operating procedure [9]

The choice of anesthesia, anesthetic agents, and type of airway devices were made by a certified anesthesiologist. The decision to give general anesthesia combined with epidural analgesia, caudal block, or peripheral nerve block was made by a certified anesthesiologist with at least 1 year experience. There were on average 18 certified anesthesiologists per year during the four-year study period. The anesthetic resident and anesthetic nurse observed children from induction until discharge from the recovery room under supervision of staff anesthesiologists. At the end of the operation, children were transferred either to the PACU or pediatric intensive care unit. Children who had SpO_2_ < 95% would receive 100% oxygen via facemask during transfer to the PACU. Children who received massive transfusion, or had potential cardiovascular or respiratory problems were transferred to the pediatric intensive care unit to continue intubation with mechanical ventilation.

At the PACU, anesthetic nurses gave an oxygen facemask (aerosol mask) via high flow air/oxygen blender with a dial setting at fractional inspired oxygen 0.4 up to 0.6. The flow rate was adjusted depending on the patient’s respiratory pattern to achieve fractional inspired oxygen at the set point. If a patient refused to wear an oxygen facemask, oxygen insufflation was performed by blowing into the patient’s nostrils and mouth. Low flow oxygen via nasal cannula is rarely used because it causes nasal discomfort and more humidity is needed. Oxygen therapy via oxygen facemask was continued at the pediatric ward depending on the discretion of the anesthesiologist based on three conditions. First, in some high-risk patients, such as those with obstructive sleep apnea syndrome or prolonged operation, oxygen therapy was not discontinued. Second, after oxygen therapy was discontinued, oxygen therapy was reinstituted in case of dyspnea, having respiratory events, or if SpO_2_ fell below 95%. Third, in case of suspected upper airway edema, the patient was endotracheally intubated with spontaneous breathing via oxygen T-piece.

### Outcome of interest

#### Postoperative oxygen therapy

Postoperative oxygen therapy was defined when patients were given postoperatively an oxygen facemask, oxygen insufflation, or oxygen T-piece at the PACU and pediatric ward. Children who did not immediately receive postoperative oxygen but required oxygen at or after postoperative day 1 or needed postoperative mechanical ventilation were excluded at the beginning of the study process and were not included in the analysis. Use of postoperative oxygen therapy was retrieved from the validated anesthetic records by two investigators (MO, SR) and confirmed by the nurses’ notes from the hospital information system. Children who were given postoperative oxygen therapy were defined as cases and each case was randomly matched with four patients who were not given postoperative oxygen therapy (controls). The four controls increased the power of the study due to the low number of cases.

#### Duration of postoperative oxygen therapy

Oxygen saturation by pulse oximetry was continuously monitored during oxygen therapy. Weaning off oxygen therapy was attempted at the pediatric ward when the patient had no signs or symptoms of respiratory disturbance, or the SpO_2_ reached 95% while the patient was breathing room air. The duration of postoperative oxygen therapy in hours was defined from the time of starting oxygen therapy at the PACU until terminating oxygen therapy and the patient could breathe spontaneously. This information was retrieved from the nurses’ notes from the electronic hospital information system. The start and stopping dates of oxygen therapy were recorded by agreement of the two investigators (MO, SR).

### Matching procedure

All cases were identified from the anesthetic records and the hospital information system according to the above-mentioned criteria. To avoid selection bias and imbalance between cases and controls, a matching algorithm was constructed. The list of controls was selected from the pool of patients kept in the department of pediatrics database. Data management with application of the exclusion criteria was managed by MO to accomplish the qualified controls. Each case was randomly matched with four qualified controls based on age group (< 1, 1–6, > 6 years) and year of operation.

### Potential predictors and confounding variables

Covariates were categorized into 11 patient-related factors, two surgery-related factors (elective/emergency and site of procedure), seven anesthesia-related factors, and occurrence of respiratory events during anesthesia. Patient-related factors included sex, body mass index (kg/m^2^), history of upper respiratory tract infection (URI) (defined as any sign or symptom of rhinitis, cough, pharyngitis, or an active or recent URI within 2 weeks), history of hyper-reactive airway (defined as a history of allergic rhinitis or asthma), history of pulmonary disease (defined as a history of pneumonia, atelectasis, or bronchiectasis within 2 weeks), history of snoring, history of non-cyanotic heart disease, history of delayed development (defined as a history of cerebral palsy or mental retardation), preoperative anemia (males and females having a hemoglobin below 13 g/dl and 12 g/dl, respectively), having a probable difficult airway, and having a risk of aspiration (defined as inadequate nothing-by-mouth time, full stomach, or history of gastroesophageal reflux) (Table 1). Anesthesia-related factors included ASA physical status, choice of anesthesia, airway device, neuromuscular blocking agent used, volatile anesthetic agent used, narcotic used, and anesthetic time (Table 2). A respiratory event during anesthesia was defined as having an event such as laryngospasm (defined as a partial or complete upper airway obstruction from a spasm of the vocal cords), upper airway obstruction (defined as soft tissue obstruction or secretion obstruction after the airway device was removed), bronchospasm (defined as a lower airway obstruction from a spasm of bronchial smooth muscle) or wheezing, or desaturation < 95% for at least 10 s [10] during the intraoperative period or at the PACU.

**Table 1:** Demographic data among children who received postoperative oxygen therapy compared with those who did not

Factor	No oxygen therapy (*n* = 1152)	Oxygen therapy (*n* = 288)	*p* value
Age (years)			0.99
<1	216 (18.8)	54 (18.8)	
1–6	552 (47.9)	138 (47.9)	
>6	384 (33.3)	96 (33.3)	
Male	684 (59.4)	182 (63.2)	0.26
Weight, median (IQR) (kg)	16.0 (9.8-25.0)	14.3 (8.4-33.4)	0.41
Height, median (IQR) (cm)	104.0 (80-128.0)	98.5 (75.0-130.0)	0.19
Body mass index (kg/m^2^)			<0.001^*^
5–14.9	479 (41.6)	130 (45.1)	
15–24.9	629 (54.6)	128 (44.4)	
25-60	44 (3.8)	30 (10.4)	
URI (Y/N)	72 (6.2)/1080 (93.8)	23 (8)/265 (92.0)	0.35
Pulmonary disease (Y/N)	8 (0.7)/1144 (99.3)	16 (5.6)/272 (94.4)	<0.001^*^
Hyper-reactive airway (Y/N)	65 (5.6)/1087 (94.4)	24 (8.3)/264 (91.7)	0.12
Anemia (Y/N)	526 (45.7)/626 (54.3)	127 (44.1)/161 (55.9)	0.68
Snoring (Y/N)	14 (1.2)/1138 (98.8)	4 (1.4)/284 (98.6)	0.95
Non-cyanotic heart (Y/N)	41 (3.6)/1111 (96.4)	30 (10.4)/258 (89.6)	<0.001^*^
Probable difficult airway (Y/N)	12 (1.0)/1140 (99.0)	21 (7.3)/267 (92.7)	<0.001^*^
Risk of aspiration (Y/N)	24 (2.1)/1128 (97.9)	11 (3.8)/277 (96.2)	0.13
Delayed development (Y/N)	34 (3.0)/1118 (97.0)	22 (7.6)/266 (92.4)	<0.001^*^

Data are presented as frequency (percentage) unless otherwise stated

^*^<0.05 by chi-squared test

*IQR* inter-quartile range, *URI* Upper respiratory tract infection

**Table 2:** Anesthesia- and surgical-related risk among children who received postoperative oxygen therapy compared with those who did not

Factor	No oxygen therapy (*n* = 1152)	Oxygen therapy (*n* = 288)	*p* value
ASA physical status			<0.001^*^
1	242 (21.0)	21 (7.3)	
2	742 (64.4)	136 (47.2)	
3	168 (14.6)	131 (45.5)	
Type of case			<0.001^*^
Elective	984 (85.4)	212 (73.6)	
Emergency	168 (14.6)	76 (26.4)	
Site/ type of procedure			<0.001^*^
Eye	226 (19.6)	11 (3.8)	
Urologic	212 (18.4)	33 (11.5)	
Airway	187 (16.2)	66 (22.9)	
Ear-nose-face	93 (8.1)	19 (6.6)	
Thoracic	2 (0.2)	23 (8.0)	
Intra-abdomen	84 (7.3)	49 (17.0)	
Orthopedic	238 (20.7)	50 (17.4)	
Intra-cranial	14 (1.2)	9 (3.1)	
Gastrointestinal scope	53 (4.6)	13 (4.5)	
Cardiac catheterization	43 (3.7)	15 (5.2)	
Choice of anesthesia			0.04^*^
GA only	948 (82.3)	252 (87.5)	
GA with epidural/caudal	151 (13.1)	31 (10.8)	
GA with peripheral nerve block	53 (4.6)	5 (1.7)	
Airway device			<0.001^*^
Facemask/laryngeal mask airway	411 (35.7)	31 (10.8)	
Spontaneous breathing with non-rebreathing mask	28 (2.4)	4 (1.4)	
Endotracheal tube intubation	713 (61.9)	253 (87.8)	
Neuromuscular blocking agent			<0.001^*^
Succinylcholine	84 (7.3)	45 (15.6)	
Aminosteroid derivatives	39 (3.4)	29 (10.1)	
Benzylisoquinolines	456 (39.6)	141 (49.0)	
None	573 (49.7)	73 (25.3)	
Inhalation agent			0.99
Sevoflurane	945 (82.0)	236 (81.9)	
Desflurane	24 (2.1)	6 (2.1)	
None	183 (15.9)	46 (16.0)	
Narcotic			0.006^*^
Fentanyl	841 (73.0)	204 (70.8)	
Morphine	100 (8.7)	42 (14.6)	
None	211 (18.3)	42 (14.6)	
Anesthetic time (hour)			<0.001^*^
<1	281 (24.4)	27 (9.4)	
1–<3	737 (64.0)	201 (69.8)	
≥3	134 (11.6)	60 (20.8)	
PRAE			<0.001^*^
None	1,130 (98.1)	129 (44.8)	
Upper airway obstruction	0 (0)	9 (3.1)	
Laryngospasm	9 (0.8)	3 (1.0)	
Desaturation	7 (0.6)	126 (43.8)	
Bronchospasm/wheezing	6 (0.5)	21 (7.3)	

Data are presented as frequency (percentage) unless otherwise stated

^*^<0.05 by chi-squared test

*ASA* American Society of Anesthesiologists, *GA* General anesthesia, *PRAE* Perioperative respiratory adverse events

### Statistical analysis

All variables are presented descriptively with mean and standard deviation or median and interquartile range as appropriate for continuous variables, and frequency and percent for categorical variables. Unpaired Student’s t-tests or Wilcoxon’s rank sum tests were used to compare normally or non-normally distributed variables, respectively, between the two outcome groups. The chi-square test or Fisher’s exact test was used to compare categorical variables. Predictors of the use and duration of postoperative oxygen therapy were determined using a hurdle model with adjustment for potential confounders [11, 12]. The hurdle model had two sets of predictors. The first set predicted whether the patient used any oxygen therapy postoperatively. The second set predicted the duration of oxygen therapy among the cases. The model was refined by sequential backward elimination of non-significant variables performed by the likelihood ratio test that provided coefficients and their 95% confidence intervals. The exponential of these coefficients gave adjusted odds ratios to assess the effects of various factors on any oxygen therapy and adjusted count ratios to assess the multiplying effects of various factors on duration (hours) of oxygen therapy. Factors were considered significant if the likelihood ratio test *p* values were < 0.05.

### Hurdle model

Hurdle models are one type of regression model for count data that can handle zero inflated outcomes, i.e., many zero observations. There are two-components in the modeling process: a truncated count component which is often modeled using a Poisson or negative binomial distribution for non-zero counts (in this study the number of hours of postoperative oxygen therapy), and a hurdle component which models the probability of a zero count, i.e., no oxygen therapy, versus non-zero count, i.e., use of oxygen. The zero count process is modeled using logistic regression. Due to over-dispersion, i.e., a situation where the variance is much larger than the mean, in the number of hours of oxygen therapy and the fact that many children in our study did not receive oxygen therapy, a negative binomial hurdle model was chosen for this analysis. This distribution also fit our data better than the Poisson model.

The hurdle model is given by the following equation:

$$ {\mathrm{f}}_{hurdle}\left(y;x,z,\beta, \upgamma \right)=\left\{\begin{array}{l}{\mathrm{f}}_{zero}\left(0;z,\upgamma \right),\kern10.6em \mathrm{if}\kern0.5em y=0\\ {}\left(1-{\mathrm{f}}_{zero}\left(0;z,\upgamma \right)\right)\times \frac{{\mathrm{f}}_{count}\left(y;x,\beta \right)}{1-{\mathrm{f}}_{count}\left(0;x,\beta \right)},\kern1em \mathrm{if}\kern0.5em y>0\end{array}\right. $$

*y* = outcome (number of hours of postoperative oxygen therapy)

*x* = potential predictors of the truncated count data model

*z* = potential predictors of the zero hurdle model

*β* = vector of regression coefficients of f_count_

*γ* **=** vector of regression coefficients of f_zero_

The regression coefficients are estimated using maximum likelihood. The corresponding mean regression relationship is given by:

$$ \mathrm{Log}\ \left({\mu}_{\mathrm{i}}\right)={x_{\mathrm{i}}}^{\mathrm{T}}\beta +\log \left\{1-{\mathrm{f}}_{\mathrm{zero}}\ \left(0;{z}_{\mathrm{i}},\upgamma \right)\right\}-\log\ \left\{1-{\mathrm{f}}_{\mathrm{count}}\left(0;{x}_{\mathrm{i}},\beta \right)\right\} $$

where *μ*_i_ = mean number of hours using oxygen.

### Risk prediction tool

The risk prediction tool was developed using the first set of predictors from the hurdle model. A test for discrimination was used to attain the predictive accuracy of the tool. The risk score of postoperative oxygen therapy was calculated from the coefficients of the significant covariates in the final model [13]. Scores were obtained by multiplying each coefficient by 2 and then rounding the result to the nearest integer for simple interpretation. Model discrimination performance was examined using the area under the receiver operating characteristic (ROC) curve providing sensitivity and specificity based on the optimal cut-point of the risk score.

### Sample size determination

We estimated the lowest prevalence of exposure (potential predictor) among the controls to be 5% with a ratio of controls to cases of 4:1 to detect an odds ratio of at least 2.0 using a significance level of 0.05 and a power of 80% that resulted in a required sample size of 250 cases and 1000 controls. The mean prevalence of postoperative oxygen therapy among non-cyanotic children in our hospital between 2010 and 2013 was 3%. Therefore, 4 years of retrospective data were required to obtain this sample size.

## Results

**Fig. 1:**
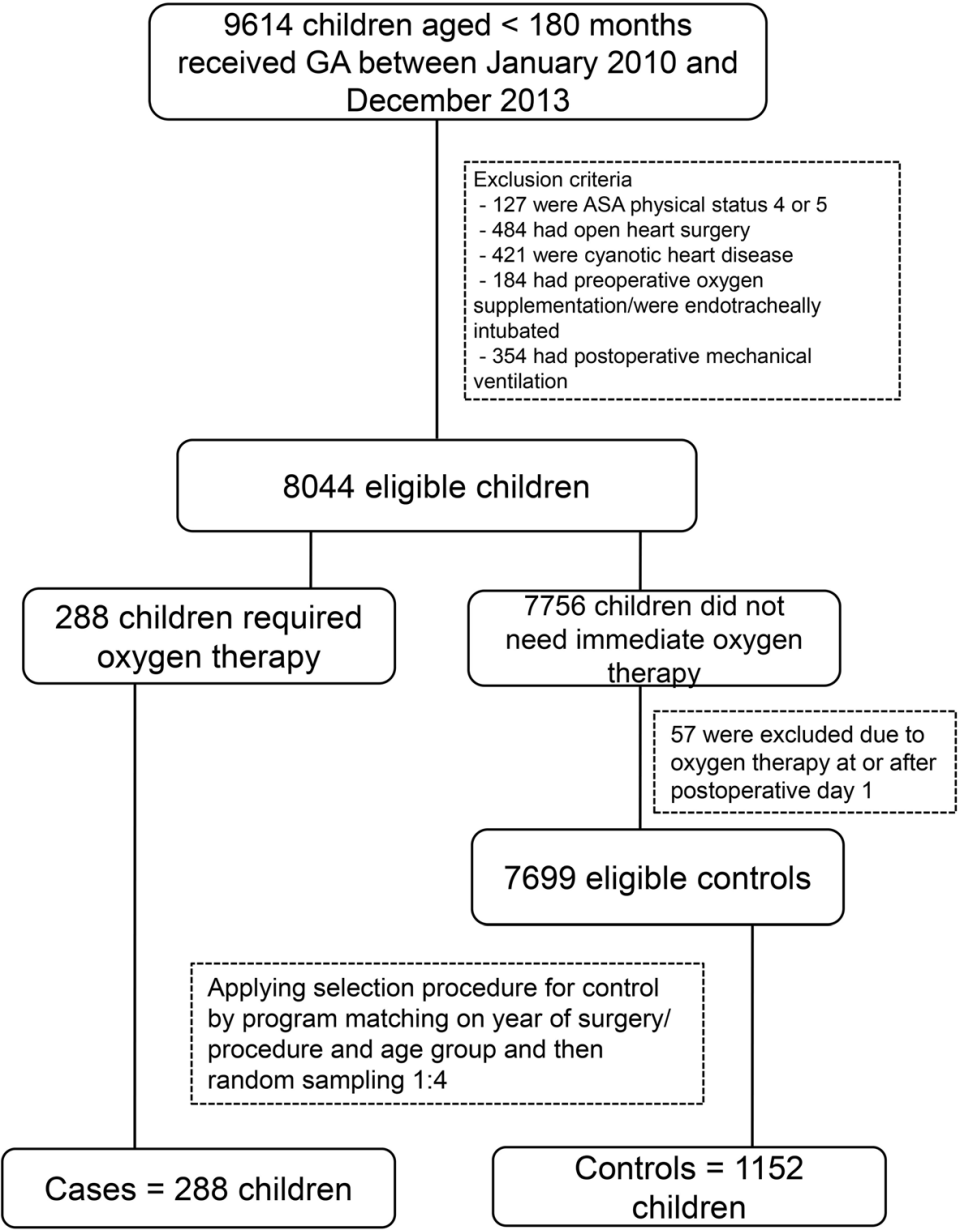
Flow diagram of the study. *GA* General anesthesia, *ASA* American Society of Anesthesiologists

From the database, 9614 children received general anesthesia during the four-year study period. After applying the exclusion criteria, 8044 children were eligible for the study. Figure 1 shows the flow diagram of the study which contained 288 cases and 1152 matched controls. Five percent of height data were missing and replaced by the mean height of children of the same age and sex. The reasons for giving postoperative oxygen therapy were prophylaxis in high risk patients or high risk operations (43.8%), perioperative desaturation (44.4%), perioperative wheezing/rhonchi (4.5%), spontaneous breathing via T-piece (3.8%), perioperative upper airway obstruction (2.4%), and shivering (1%). Some children (< 50%) who had either a probable difficult airway or airway surgery were endotracheally intubated and received oxygen via the T-piece connector. Table 1 compares the demographic data of children who received postoperative oxygen therapy to those who did not. High body mass index, history of pulmonary disease, history of non-cyanotic heart disease, having a probable difficult airway, and history of delayed development were significantly different between the two groups. Table 2 shows a similar comparison of anesthesia-related and surgery-related risk factors. There were significant differences between the two groups in terms of ASA physical status, elective/emergency case, site and type of surgery, choice of anesthesia, airway device, neuromuscular blocking agents used, narcotic used, anesthetic time, and having a respiratory event during anesthesia.

### Analysis of postoperative oxygen therapy

Seven variables having a *p* value ≤ 0.2 from the univariate analysis were included in the initial multivariate hurdle model but were not significant: history of pulmonary disease, history of non-cyanotic heart disease, history of delayed development, having risk of aspiration, elective/emergency, choice of anesthesia, and narcotic used. Table 3 shows the results of the final hurdle model predicting postoperative oxygen therapy.

**Table 3:** Multivariate hurdle model of factors predicting postoperative oxygen therapy use and risk scores in children undergoing non-cardiac surgery

Factor	Coefficient	Adjusted OR (95% CI)	*p* value	Risk score
Body mass index (Ref: 15-24.9)				
5–14.9	0.51	1.66 (1.07, 2.58)	0.024	1
25-60	1.04	2.82 (1.30, 6.11)	0.009	2
Hyper-reactive airway (Ref: No)				
Yes	0.92	2.50 (1.12, 5.59)	0.025	2
Probable difficult airway (Ref: No)				
Yes	1.61	4.99 (1.74, 14.32)	0.003	3
ASA physical status (Ref: 1)				
2	0.31	1.36 (0.73, 2.53)	0.327	0
3	1.55	4.73 (2.43, 9.18)	<0.001	3
Site/ type of procedure (Ref: Eye)				
Urologic	1.46	4.32 (1.39, 13.41)	0.01	3
Airway	2.04	7.72 (2.62, 22.70)	0.0002	4
Ear-nose-face	0.82	2.27 (0.60, 8.52)	0.22	0
Thoracic	5.63	277.9 (44.56, 1733)	<0.0001	11
Intra-abdomen	1.96	7.12 (2.30, 22.04)	0.0007	4
Orthopedic	1.95	7.00 (2.30, 21.33)	0.0006	4
Intra-cranial	2.01	7.45 (1.62, 34.20)	0.01	4
Gastrointestinal scope	1.23	3.42 (0.88, 13.37)	0.07	0
Cardiac catheterization	1.98	7.28 (2.04, 25.96)	0.002	4
Airway device (Ref: Facemask/LMA)				
Spontaneous breathing with non- rebreathing mask	1.38	3.98 (0.64, 24.88)	0.14	0
Endotracheal tube intubation	1.89	6.64 (2.54, 17.32)	0.0001	4
Neuromuscular blocking agent (Ref: None)				
Succinylcholine	0.67	1.95 (0.92, 4.13)	0.080	0
Aminosteroid derivatives	1.06	2.89 (1.18, 7.05)	0.02	2
Benzylisoquinolines	0.07	1.07 (0.58, 1.96)	0.83	0
Anesthetic time (hours) (Ref: <1)				
1–2.9	1.09	2.99 (1.38, 6.44)	0.005	2
≥3	1.57	4.79 (1.99, 11.6)	0.0005	3
PRAE (Ref: None)				
UAO and laryngospasm	4.12	61.5 (14.4, 262)	<0.0001	8
Desaturation	6.18	481.2 (177.9, 1302)	<0.0001	12
Bronchospasm/wheezing	4.53	92.4 (29.7, 288)	<0.0001	9

*Ref* Reference group; *OR* Odds ratio, *CI* Confidence interval, *ASA* American Society of Anesthesiologists, *LMA* Laryngeal mask airway, *UAO* Upper airway obstruction, *PRAE* Perioperative respiratory adverse events

### Development of risk prediction tool

**Fig. 2:**
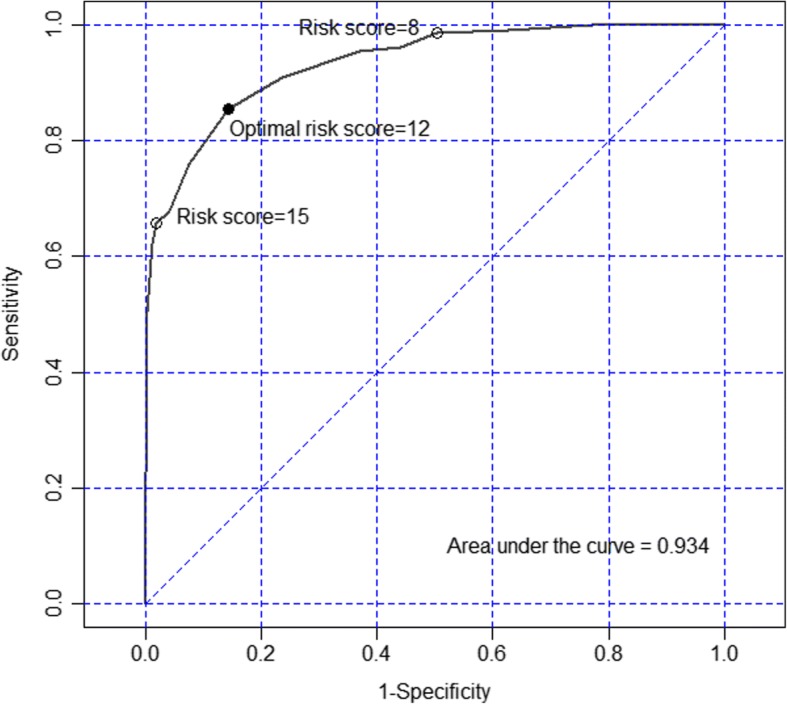
Receiver operating characteristic curve of the risk score predicting postoperative oxygen therapy

Risk scores from the model are shown in Table 3. We selected the reference group based on the subgroup which had a lowest risk. The scores were summed to obtain an individual risk score which ranged from 0 to 32. Figure 2 shows the ROC curve of the individual risk scores predicting oxygen therapy. The area under the curve was 0.93 and the optimal cut-point based on the highest summation of sensitivity (85%) and specificity (86%) of the model was 12. The risk scores were then classified into three groups: high (≥ 12), intermediate (8–11), and low (≤ 7) that indicated the level of need for postoperative oxygen therapy.

### Duration of postoperative oxygen therapy

The median (inter-quartile range) duration of oxygen therapy among the cases was 17 h (range 9–22 h). The distribution of the number of hours of oxygen use was over-dispersed, thus using a negative binomial hurdle model fit the data much better than the Poisson hurdle model (Additional file 1). Nine variables that had a *p* value ≤0.2 in the univariate analysis were included in the initial multivariate hurdle model but were not significant: body mass index, history of URI, history of pulmonary disease, history of snoring, anemia, choice of anesthesia, airway device, neuromuscular blocking agent used, and having a respiratory event during anesthesia (Additional file 2). Table 4 shows the results of the final hurdle model predicting duration of postoperative oxygen therapy.

**Table 4:** Multivariate hurdle model of factors predicting duration of postoperative oxygen therapy use in children undergoing non-cardiac surgery

Factor	Adjusted count ratio (95% CI)	*p* value
Probable difficult airway	2.19 (1.39, 3.48)	0.0008
History of delayed development	2.31 (1.49, 3.57)	0.0002
ASA physical status (Ref: 1)		0.03^a^
2	0.75 (0.43, 1.32)	
3	1.07 (0.61, 1.88)	
Site/type of procedure (Ref: Eye)		<0.0001
Urologic	1.98 (0.99, 3.96)	
Airway	3.01 (1.55, 5.83)	
Ear-nose-face	2.27 (1.06, 4.82)	
Thoracic	4.86 (2.34, 10.1)	
Intra-abdomen	4.15 (2.13, 8.11)	
Orthopedic	2.14 (1.08, 4.25)	
Intra-cranial	1.26 (0.52, 3.02)	
Gastrointestinal scope	2.73 (1.23, 6.04)	
Cardiac catheterization	1.18 (0.53, 2.60)	
Narcotic used (Ref: None)		0.049^b^
Morphine	0.69 (0.40, 1.20)	
Fentanyl	1.10 (0.72, 1.68)	

*Ref* Reference group, *ASA* American Society of Anesthesiologists

^a^The contrast of ASA physical status 3 vs ASA physical status 2 gave a significant count ratio of 1.42 and 95% CI of 1.10 and 1.84.

^b^The contrast of morphine vs fentanyl gave a significant count ratio of 0.63 and 95% CI of 0.44 and 0.90.

## Discussion

This matched case-control study determined predictors of the use and duration of postoperative oxygen therapy using a hurdle model to present two sets of predictors. A risk prediction tool for postoperative oxygen therapy was developed giving an area under the ROC curve of 0.93. Using the risk scores obtained from the model coefficients, children were classified into high (score ≥ 12), intermediate (8–11), or low (≤7) risk groups. These risk scores can be used to determine the level of need for postoperative oxygen therapy among children with similar risk factors.

### Risk prediction tool for postoperative oxygen therapy

The optimal cut-point from the ROC curve was 12; therefore, we classified children with a score of ≥12 as high risk. A score of 8 showed high sensitivity (98.6%) which was consistent with a very low false negative rate (1.4%) (Fig. 2). Therefore, we considered a risk score < 8 to be a low risk (negative test) with a chance to require postoperative oxygen therapy < 1.4%. Thus, an individual risk score between 8 and 11 was classified as intermediate risk.

#### High risk group

The high risk group (score ≥ 12) included those who had thoracic surgery or had desaturation perioperatively. Children undergoing thoracic surgery (score of 11) usually need to be intubated with an endotracheal tube (score of 4) and are at high risk for postoperative oxygen therapy because they have a decrease of pulmonary functional residual capacity and high disturbance of cardiopulmonary function [14–16]. Provision of oxygen supplementation at the PACU and at a ward is necessary to prevent pulmonary complications. Children who had perioperative desaturation were also classified as high risk. Strategies to identify risk or develop a risk prediction tool to predict the occurrence of perioperative respiratory events would help to reduce the need for postoperative oxygen therapy [9, 13, 17, 18].

#### Intermediate risk group

The intermediate risk group (score of 8–11) included those who had bronchospasm, upper airway obstruction or laryngospasm perioperatively, or from a combination of patient risk factors, such as high body mass index (score of 2), history of hyper-reactive airway (score of 2), having probable difficult airway (score of 3), other types of operations (score of 0–4), and anesthesia risk factors, such as ASA physical status 3 (score of 3), endotracheal tube intubation (score of 4), and anesthetic time ≥ 1 h (score of 2–3).

Obesity was found to be associated with the limitation of functional residual capacity and cardiovascular comorbidities that caused desaturation [13, 19–21] which may necessitate postoperative oxygen therapy. A child with a hyper-reactive airway (due to asthma) is prone to develop bronchospasm or desaturation due to airway hypersensitivity to stimuli during anesthesia. Having a probable difficult airway [22, 23] and prolonged anesthetic time [19, 24] may lead to the need for postoperative oxygen therapy due to upper airway obstruction, desaturation, and perioperative reintubation. The ability to quickly and easily calculate a risk score for children undergoing non-cardiac surgery might help anesthesia personnel make decisions concerning the child’s management both at the preoperative and intraoperative periods. For example, if a child has an individual risk score of 6 or 7, the health personnel may try to reduce this score by preventing desaturation in difficult airway cases or by avoiding both endotracheal tube intubation and long or intermediate use of neuromuscular blocking agents intraoperatively, if feasible.

### Predictors for duration of postoperative oxygen therapy

The median duration of postoperative oxygen therapy in our study was 17 h which is a duration that is quite safe from the harmful effects of oxygen therapy in children. The significant predictors for longer use of oxygen therapy included: history of delayed development, having probable difficult airway, thoracic, abdominal, airway and orthopedic surgery, and ASA physical status 3.

Having a probable difficult airway and airway surgery were predictors for both postoperative oxygen therapy and duration of use. Most of the children who were given oxygen via the T-piece connector (*n* = 11) had a probably difficult airway and airway surgery, thus they had a longer duration of oxygen therapy since duration (in hours) included the timing from receiving oxygen T-piece, removing endotracheal tube to weaning oxygen therapy via facemask. Children with cerebral palsy and mental retardation, defined as having delayed development, was another strong predictor for longer use of oxygen therapy (count ratio = 2.3). Most children with delayed development have several comorbidities, including cognitive disability, and have a higher risk of respiratory problems such as lower airway infection, airway secretion, or aspiration which may require longer use of postoperative oxygen therapy [25].

Compared with eye surgery, which had the lowest risk for postoperative oxygen therapy, ear-nose-face surgery and gastrointestinal endoscopy were also predictors for longer use of oxygen therapy. Ear-nose-face surgery cases included fractures of the face bones which necessitated postoperative endotracheal intubation and oxygen therapy via the T-piece connector, while children who had gastrointestinal endoscopy were more likely to have a history of delayed development. Use of morphine decreased the duration of postoperative oxygen therapy by a factor of about two-thirds compared to fentanyl. In this study, morphine was usually given (> 80%) in both older children (> 10 years) and those with ASA physical status 1 or 2 which may result in earlier discontinuation of postoperative oxygen therapy compared to those who received fentanyl. However, since age group was matched between cases and controls and ASA physical status was adjusted in the final model (Table 4), an explanation of this result is inconclusive as no other study has reported a similar result. Further studies focusing on opioids for postoperative oxygen therapy may help to explain this result.

Since 80% of respiratory events during anesthesia were mild desaturation (SpO_2_ 90–94%) and brief in duration (< 60 s), which required oxygen therapy for a short duration (< 17 h), it was not a predictor for duration of oxygen therapy. Thus, low flow oxygen or a lower oxygen concentration should be considered for those at high risk to reduce the risk of oxygen toxicity [26, 27]. Therefore, we attempted to limit the fraction of inspired oxygen between 0.4 and 0.6. We also provided continuous oxygen saturation monitoring and weaned off the oxygen therapy as soon as possible in almost 25% of the children who received oxygen therapy for more than 24 h.

### Important issues and implications for clinical management

Our risk prediction tools have implications for aiding physicians in their clinical decision making peri-operatively. These can be divided into four strategic steps.

First, pre-operatively, physicians can utilize the risk prediction tool to quickly calculate a risk score indicating the degree of need for post-operative oxygen therapy. For example, an obese child (score + 2), or one with hyperactive airway (+ 2), history of difficult airway (+ 3), or with an ASA physical status 3 (+ 3), would have an increased risk of requiring post-operative oxygen therapy. Having multiple risk factors would also increase the requirement substantially.

Second, combining pre-operative with intra-operative risk factors can help physicians determine which children have an increased need for post-operative oxygen therapy, for example, children who undergo thoracic surgery or cardiac catheterization. In addition, children with a history of delayed development or those requiring a gastrointestinal scope may not only require post-operative oxygen therapy but may require prolonged use.

Third, children who score highly (≥ 12) on pre- and intra-operative risk factors can be targeted for receiving extra attention by anesthetic personnel. On the other hand, children who have low scores (≤ 7) may be given short-acting neuromuscular blocking agents, or given an anesthetic device that has a low risk of oxygen therapy postoperatively such as a laryngeal mask airway or facemask, or given shorter anesthetic time (if feasible), or tried to minimize the incidence of respiratory events during anesthesia.

Fourth, minimizing the risk of prolonged oxygen therapy, by minimizing the risk score, should also result in a reduced duration of postoperative oxygen therapy. If oxygen therapy is absolutely necessary, for example when given as a prophylaxis in high risk children or when a respiratory event occurs during anesthesia, weaning children off oxygen therapy should be considered in those who have a low (≤ 7) or intermediate (8–11) risk score as soon as possible when signs and symptoms of hypoxia have improved in order to minimize the consequences of excessive use of postoperative oxygen therapy [28, 29]. It has been suggested that an audit of the criteria used for prescribing oxygen such as target saturations, oxygen device used, and indication for use, would help wean children off postoperative oxygen therapy [30]. However, if the prolonged need for postoperative oxygen therapy cannot be avoided, the lowest fraction of inspired oxygen concentration (< 0.5) should be considered to prevent long term complications and enhance patient safety.

### Strengths and limitations

This study has some strengths. Matching cases to controls was done using age groups and year of surgery to reduce selection bias and to balance the two groups in terms of age. The multivariate hurdle model to predict both outcomes (any use and duration of postoperative oxygen therapy) was performed to adjust for potential confounding variables. The sample size was adequate which resulted in a high area under the ROC curve (0.93).

Even though we attempted to minimize the selection bias, some information bias from the database could have occurred. The incidence of a respiratory event during anesthesia in our study as well as some patient-related factors such as snoring and URI were quite low compared to a previous prospective study [24]. Finally, the child’s age, which might affect the need for postoperative oxygen therapy, could not be evaluated since it was used for matching.

Even though this was a retrospective study, the reliability and accuracy of our results are quite high because the outcomes were determined from records confirmed by two experienced anesthesiologists, the study was matched on age and year of operation, and the sample size was large. The external validity to other children in the same setting should be convincing although the subjects were recruited from a single university hospital.

## Conclusions

Our risk prediction tool for postoperative oxygen therapy provided a high predictive ability and severity of risk prediction score. For both outcomes, the common risk factors were: probable difficult airway, ASA physical status 3, and operations involving the airway, thorax, and abdomen. Anesthesia-related and surgery-related factors played an important role in the longer use of postoperative oxygen therapy. Some patient-related and surgery-related risks may not be preventable but the adjustment of oxygen therapy can be made under patient safety issues.

## Additional files

Additional file 1: The distribution of the number of hours of oxygen use. (TIFF 1009 kb)

Additional file 2: Univariate hurdle model predicting duration of postoperative oxygen therapy use in children undergoing non-cardiac surgery (*n* = 1440). (PDF 43 kb)

## Abbreviations

ASA: American Society of Anesthesiologist
PACU: Post-anesthetic care unit
ROC: Receiver operating characteristic
URI: Upper respiratory tract infection
SpO_2_: Oxygen saturation by pulse oximetry
