# Supplementary figures and images for: Risk prediction tool for use and predictors of duration of postoperative oxygen therapy in children undergoing non-cardiac surgery: a case-control study
Source: BMC Anesthesiol. 2018 Nov 2;18:137. doi: 10.1186/s12871-018-0595-4 (PMC6214164; doi:10.1186/s12871-018-0595-4)

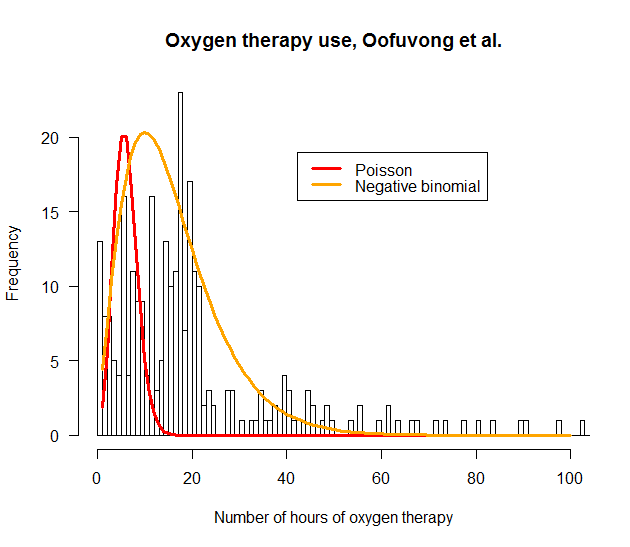

Supplement: Supplementary file 1 — The distribution of the number of hours of oxygen use. (TIFF 1009 kb) [file 12871_2018_595_MOESM1_ESM.tiff]
