# Supplementary material for: Risk prediction tool for use and predictors of duration of postoperative oxygen therapy in children undergoing non-cardiac surgery: a case-control study
Source: BMC Anesthesiol. 2018 Nov 2;18:137. doi: 10.1186/s12871-018-0595-4 (PMC6214164; doi:10.1186/s12871-018-0595-4)
Supplement: Supplementary file 2 — Univariate hurdle model predicting duration of postoperative oxygen therapy use in children undergoing non-cardiac surgery (n = 1440). (PDF 43 kb) [file 12871_2018_595_MOESM2_ESM.pdf]

Additional file 2 Univariate hurdle model predicting duration of postoperative oxygen therapy use in children undergoing non-cardiac surgery (n=1440)

Variables	Count ratio (95% CI)	p value
Body mass index (Ref: 15-24.9)		0.063
5-14.9	1.33 (1.01, 1.76)	
25-60	1.55 (0.98, 2.45)	
Upper respiratory tract infection (Ref: No) Yes	0.54 (0.33, 0.88)	0.02
Pulmonary disease (Ref: No) Yes	1.89 (1.06, 3.37)	0.006
Hyper-reactive airway (Ref: No)Yes	0.91 (0.56, 1.49)	0.74
Anemia (Ref: No) Yes	1.22 (0.93, 1.59)	0.16
Snoring (Ref: No) Yes	0.44 (0.14, 1.40)	0.17
Non-cyanotic heart disease (Ref: No) Yes	0.88 (0.57, 1.37)	0.37
Probable difficult airway (Ref: No) Yes	2.08 (1.26, 3.44)	0.006
Risk of aspiration (Ref: No) Yes	1.05 (0.52, 2.11)	0.61
History of delayed development (Ref: No) Yes	2.37 (1.45, 3.85)	<0.001
ASA physical status (Ref: 2)		<0.001
1	0.85 (0.51, 1.44)	
3	1.69 (1.29, 2.21)	
Emergency case (Ref: Elective)	0.9 (0.66, 1.21)	0.49
Site of procedure (Ref: Eye)		<0.001
Urologic	1.70 (0.82, 3.53)	
Airway	3.57 (1.80, 7.08)	
Ear-nose-face	2.52 (1.14, 5.57)	
Thoracic	4.93 (2.29, 10.58)	
Intra-abdomen	4.08 (2.03, 8.21)	
Orthopedic	1.58 (0.79, 3.19)	
Intra-cranial	1.21 (0.47, 3.11)	
Gastrointestinal scope	2.05 (0.87, 4.83)	
Cardiac catheterization	1.60 (0.70, 3.68)	
Choice of anesthesia (Ref: GA only)		0.007
GA with epidural/caudal	0.66 (0.43, 1.01)	
GA with peripheral nerve block	0.18 (0.06, 0.52)	
Airway device (Ref: Facemask/ LMA)		0.01
Spontaneous breathing with non-rebreathing mask	1.19 (0.36, 3.92)	
Endotracheal tube intubation	1.98 (1.29, 3.05)	
Neuromuscular blocking agent (Ref: None)		0.02
Succinylcholine	1.43 (0.94, 2.18)	
Aminosteroid derivatives	1.03 (0.63, 1.68)	
Benzylisoquinolines	1.67 (1.21, 2.30)	
Narcotic used (Ref: Fentanyl)		0.03
Morphine	0.60 (0.41, 0.87)	
None	0.70 (0.48, 1.02)	
Anesthetic time (hour) (Ref: <1)		0.87
1 - <3	1.16 (0.73, 1.86)	
≥3	1.07 (0.64, 1.83)	
Respiratory events during anesthesia (Ref: None)		0.062
Upper airway obstruction and laryngospasm	2.04 (1.04, 4.00)	
Bronchospasm/wheezing	0.85 (0.50, 1.43)	
Desaturation	0.86 (0.65, 1.14)	

p-value by Likelihood ratio test, ASA= American Society of Anesthesiologists;
CI=Confidence interval; GA=General anesthesia
